# *Theileria parva* in Zambia: A scoping review protocol

**DOI:** 10.1371/journal.pone.0341896

**Published:** 2026-02-23

**Authors:** Chishimba M. Mubanga, Choolwe Malabwa, Noreen Machila, Martin C. Simuunza

**Affiliations:** 1 Department of Disease Control, School of Veterinary Medicine, The University of Zambia, Lusaka, Zambia; 2 Central Veterinary Research Institute, Ministry of Fisheries and Livestock, Lusaka, Zambia; 3 Africa Centre of Excellence for Infectious Diseases of Humans and Animals, University of Zambia, Lusaka, Zambia; Freie Universität Berlin, GERMANY

## Abstract

**Objective:**

The objective of this scoping review is to examine published and unpublished literature on *Theileria parva* in Zambia. It will identify the scope of available literature for research prioritization.

**Introduction:**

*Theileria parva* is the most lethal tick borne disease in Zambia due to its reportedly high morbidity and mortality in cattle. It is a focus of government interventions. It is important to understand the research that has been conducted on this parasite over the years.

**Inclusion criteria:**

The review will include all studies conducted on *Theileria parva* in Zambia. Government and research institute reports based on recognized laboratory diagnosis will also be included.

**Methods:**

Literature search will be conducted in Pubmed, Scopus, Web of knowledge and Embase. Primary studies from 1920 to date will be included. Titles, abstracts and full text will be examined to determine eligibility for inclusion. Extracted data will be charted into a data extraction excel file by two independent reviewers. The results will be summarised and presented as a narrative summary, tables and charts.

## Introduction

Livestock diseases are a major constraint to cattle and small ruminant production in Zambia [1]. East Coast fever (ECF), caused by *Theileria parva* (*T. parva*), was the second most prevalent disease that affected eight of the ten Zambian provinces [2], and 19.3% livestock keeping households in Zambia in 2023 [1]. As such, *Theileria Parva* (*T. parva*) infections are significant in Zambia because they are endemically responsible for high morbidity and mortalities (Makala et al., 2003; Nambota et al., 1994), and are therefore the focus of government interventions and livestock movement regulations (DVS, 2022).

Regional economic losses to ECF have been estimated at 1 million cattle/year estimated at nearly $300 million [3]. In Zambia, ECF losses are due to morbidity and mortality. The Southern province of Zambia is estimated to have lost nearly 800,000 cattle, valued at $238 million, between 1980 and the 1990s, predominantly attributed to ECF. In 1991, of the 17,426 ECF cases, 8717 ended up in mortality. Similarly, in 1992, of the 14,594 reported ECF cases, 6903 ended up in mortality. Morbidity losses are mainly due to loss in milk production from sick animals, loss in beef and manure production [2].

*Theileria parva* was first reported in Zambia in 1922, with a significant incidence of disease after 1946. Intensified integrated control interventions were undertaken, including vector control, chemotherapy, stock movement regulation, and, later on, immunization, which was undertaken between 1983 and 1986 [4] using the Muguga cocktail from East Africa. After the utilisation of foreign *T. parva* strains from Kenya in Zambia, the disease “spread very rapidly, overflowing beyond its original borders” [4]. This phenomenon, where *T. parva* vaccine components are transmitted to unvaccinated animals have been reviewed [5]. The increased transmission is sometimes, associated with ECF outbreak. This was observed when Muguga cocktail-immunised cattle were imported into the Comoros island leading to an outbreak of ECF in naive local cattle [6]. As such, understanding the immunogenicity and cross protection potential of local *T. parva* strains is crucial [7–9].

East Coast fever (ECF) immunisation is largely based on understanding the host – parasite interactions, particularly the humoral immune response between local *T. parva* strains and cattle [7,9,10]. This localized approach calls for a periodic review of all research on *T. parva* in order to identify emerging trends, research gaps and recent developments [11]. Since *T. parva* was identified in Zambia in 1922, to the best of our knowledge, there has just been one review focused on ECF in Zambia [4] conducted over 30 years ago, and another broader review covered major tick borne diseases, including ECF [12].

### Review question

The purpose of this scoping review is to consolidate all available information on *T. parva* infectious in Zambia. What is the scope of published peer reviewed and gray literature on *T. parva* in Zambia?

## Methods

### Inclusion criteria

#### Type of participants.

This review will include all studies covering any aspect of *Theileria parva* in and of Zambian origin affecting or infecting any animal species as well as farmer perceptions and socio-economic aspects of the disease.

#### Concept.

The main concept is *Theileria parva*. As such, any study that was conducted measuring any aspect of a Zambian *T. parva* will be included from 1922 when the disease was first reported in the country.

#### Context.

The review will be conducted to summarise all research undertaken about *Theileria parva* in Zambia including but not limited to strains, occurrence, vectors, hosts, immunology, epidemiology, control and prevention, economics and societal costs, farmer perceptions and experience as well as control programs.

#### Types of evidence sources.

The types of evidence sources will include published primary research studies, theses, reviews, and reports.

#### Search strategy.

The search strategy follows a four-step strategy, three steps recommended by the Joana Brigs Institute (JBI) [13] and an additional step to search for grey literature, reports from the Department of Veterinary Services, Private and University Laboratories and theses as well as references from the reference lists of included studies. Key words for *Theileria parva* were collected from electronic database indices and a search word was made primarily for PUBMED and the Boolean operators will be varied according to the different databases being searched. A preliminary initial search was carried out in PUBMED (72), Web of knowledge (99) and Embase (82) and the results compared (as indicated in the brackets) in order to test the sensitivity of the search word in December, 2025.

The search word is: (*Theileria parva parva* OR *Theileria parva lawrencei* OR Theileriasis OR Theileriosis OR East Coast fever OR Corridor disease OR January disease) AND (Zambia OR Northern Rhodesia) (S1). The search will be conducted in the first week of January, 2026.

### Study records

#### Data management.

All downloaded references will be uploaded in Mendeley Reference Manager for removal of duplicates (Deduplication). The references will be exported and uploaded in Rayyan. Further deduplication will be performed in Rayyan. Title and abstract screening as well as full text screening will be performed by two independent people (CMM and CM) in blind mode in Rayyan. In case of disagreement, the reviewers will resolve first through discussion. If not resolved, then a third reviewer will break the tie. Data will be extracted from the included studies.

#### Selection process.

The selection of the sources of evidence will be described and summarised using a chart according to the Preferred Reporting Items for Systematic Reviews and Meta-Analysis extension for Scoping reviews (PRISMA-ScR) [14].

#### Data collection process.

The data collection form will be piloted for the variety of included studies and modified accordingly. Data will be collected by one person (CMM) and a sample verified by another (CM).

#### Data items.

The charting of results will be done using the JBI data template with modifications [15]. A data charting form has been created to capture the following information; Author, date, title, journal, volume, issue, pages, study objectives, population, concept, context, type of evidence source, study design, study area, study participants, outcomes measured and key findings that relate to the review question.

### Analysis of evidence

The extracted evidence will largely be qualitative and so the analysis will be mainly descriptive. Studies will be grouped in themes and according to type of studies as previously done in a scoping review on Leishmaniasis [16].

### Timelines

Literature search will be conducted in the first week of January, 2026 and collection of grey literature will continue for the first quarter of 2026. The final manuscript is scheduled for submission in the last quarter of 2026.

### Presentation of the results

The study will present evidence pertaining to *T. parva* strains, the host range, the intermediate hosts (Vectors), the epidemiology (spatial, temporal, molecular), risks, immunology, clinical signs, treatment, immunization and other recorded control measures, economics and societal costs, farmer perceptions and attitudes, and any other information describing *T. parva* in Zambia. The study will be reported according to the PRISMA-ScR format (S2).

## Discussion

This study protocol is an undertaking to consolidate research on one of the most important livestock diseases in the Zambia, and could be the most comprehensive literature review as it combines published peer reviewed research and grey literauture from government sources, international organizations and laboratories. This is important to understand what is known and what is not known.

Despite the promise of being comprehensive, the study has potential shortcomings mainly due to the inclusion criteria. Potential inaccessibility of some reports especially from international organizations, journal articles not in English and removal of conference abstracts will lead to loss of some data. Despite these shortcomings, the accessible sources are sufficient to provide a full picture on *T. parva* in Zambia.

It is our considered conclusion that this subject remains important and worth the effort of a comprehensive review of literature.

## Supporting information

S1 File Search strings. (DOCX)

S2 File Checklist. Preferred Reporting Items for Systematic reviews and Meta-Analyses extension for Scoping Reviews (PRISMA-ScR) checklist.
(DOCX)
