# Supplementary material for: Theileria parva in Zambia: A scoping review protocol
Source: PLoS One. 2026 Feb 23;21(2):e0341896. doi: 10.1371/journal.pone.0341896 (PMC12928389; doi:10.1371/journal.pone.0341896)
Supplement: S1 File — (DOCX) [file pone.0341896.s001.docx]

**S1: Search word and string**

**Theileria parva in Zambia: A scoping review**

**Search word**

(Theileria parva OR Theileriasis OR Theileriosis OR "Corridor disease" OR "East coast fever" OR "January disease") AND (Zambia OR "Northern Rhodesia")

**Search string**

**PubMed**

("theileria parva"[MeSH Terms] OR ("theileria"[All Fields] AND "parva"[All Fields]) OR "theileria parva"[All Fields] OR ("theileriasis"[MeSH Terms] OR "theileriasis"[All Fields]) OR ("theileriasis"[MeSH Terms] OR "theileriasis"[All Fields] OR "theileriosis"[All Fields]) OR "Corridor disease"[All Fields] OR "East coast fever"[All Fields] OR "January disease"[All Fields]) AND ("zambia"[MeSH Terms] OR "zambia"[All Fields] OR "zambia s"[All Fields] OR "Northern Rhodesia"[All Fields])
